# Paraneoplastic Dermatomyositis with Carcinoma Cervix: A Rare Clinical Association

**DOI:** 10.1155/2014/836246

**Published:** 2014-12-18

**Authors:** Sumir Kumar, B. B. Mahajan, Sandeep Kaur, Amarbir Singh

**Affiliations:** ^1^GGS Medical College & Hospital, Sadiq Road, Faridkot, Punjab 151203, India; ^2^Skin OPD, GGS Medical College & Hospital, OPD Block, 1st Floor, Sadiq Road, Faridkot, Punjab 151203, India

## Abstract

Dermatomyositis is an uncommon inflammatory myopathy associated with cutaneous manifestations. It may also occur as paraneoplastic syndrome associated with various malignancies, most common of which being lung, breast, stomach, rectum, kidney, or testicular cancer. A postmenopausal woman presented to us with generalized itching along with skin rash and proximal muscle weakness of 2 years' duration. Examination revealed heliotrope rash and mechanic hands and muscle power 2/5 in proximal muscle groups of both upper and lower limbs. A clinical diagnosis of dermatomyositis was made which was supported by raised lactate dehydrogenase levels and skin biopsy findings. Past history was significant for vaginal discharge and bleeding per vagina. Further work-up revealed carcinoma cervix and she was referred to oncology department for further management. Temporal relationship and improvement of muscle weakness with treatment of underlying neoplasm supported its paraneoplastic nature. So, final diagnosis of keratinizing squamous cell carcinoma of cervix with paraneoplastic dermatomyositis was made. A nationwide cohort study of 1,012 patients with dermatomyositis in Taiwan revealed only 3 patients with cervical cancer. So this case is being reported for its rare association with carcinoma cervix and to highlight the need of detailed evaluation for underlying malignancies in patients with dermatomyositis.

## 1. Introduction

Dermatomyositis (DM) is a multisystem disorder characterized clinically by muscle weakness and cutaneous rash. It has a well-recognized relationship with various types of cancers, most common of which being lung, breast, female genital tract, stomach, rectum, kidney, or testis cancer. Dermatomyositis precedes the neoplasm in 40%; both conditions may occur together (26%) or the neoplasm may occur first (34%). The incidence of carcinoma in association with DM varies from 15 to 34% [1].

A nationwide cohort study of 1,012 patients with DM revealed only 3 patients with cervical cancer [2]. We here present a case of cervical carcinoma with paraneoplastic DM for its rarity.

## 2. Case Report

A 65-year-old nonhypertensive, nondiabetic woman presented with generalized itching since 2 years. She developed rash on body with photo aggravation. She complained of muscle weakness for the last 2 years for which she had been taking treatment from a local doctor without being investigated. She had difficulty in standing from sitting position and climbing up the stairs as well as combing her hair. There was no history of dysphagia, oral ulcers, or joint pains. She had vaginal discharge for the last 6 months and vaginal bleeding of few days duration. She was nonalcoholic and nonsmoker. She achieved menopause 15 years back.

**Figure 1:**
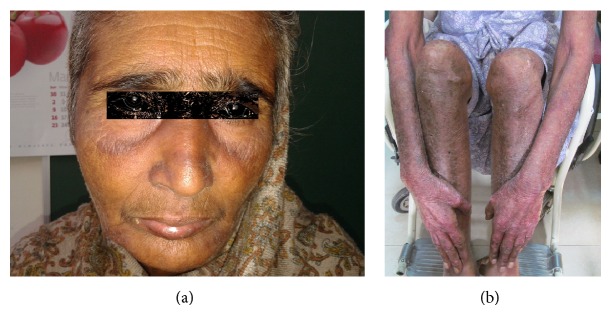
(a) Heliotrope rash in periorbital region. (b) Poikilodermatous changes evident on dorsolateral aspects of bilateral forearms.

Examination revealed presence of heliotrope rash around eyelids characterized by periorbital, confluent, and violaceous erythema (Figure 1(a)). Poikilodermatous changes were evident on V-area of neck and dorsolateral aspects of bilateral forearms and shins (Figure 1(b)). Trunk was relatively spared. On examination of hands, hyperkeratotic lesions were seen predominantly involving the centre of palms (mechanics hands) along with presence of Gottron's papules on proximal interphalangeal joints. Muscle power was 2/5 in both upper and lower limb proximal muscle groups. Per vaginal examination revealed an exfoliative growth extending into upper half of vagina. On per speculum examination, growth was seen in vagina protruding out of external cervical os. About 5 × 6 cm mass at the level of cervix with involvement of bilateral parametrium just short of pelvic wall was felt on per rectum palpation. Clinical findings strongly suggested the possibility of underlying genital tract malignancy, so oncology consultation was sought.

Results of investigations showed Hb = 7.8 g/dL, total leukocyte count = 5.25 × 10^3^/*μ*L, LDH = 960 IU/L, s. creatine kinase = 18 IU/L, and negative ANA and anti-dsDNA.

**Figure 2:**
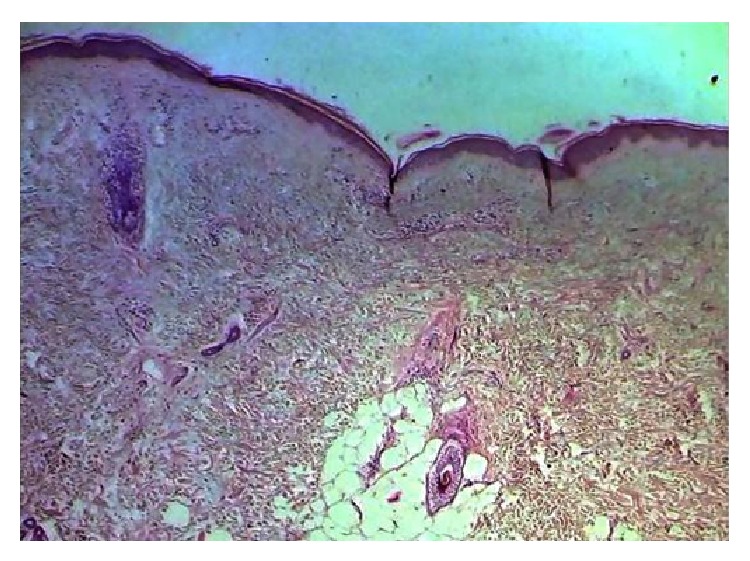
Histopathology of skin: focal thinning of epidermis along with hydropic degeneration of the basal keratinocytes. Deposition of eosinophilic material was seen particularly at dermoepidermal junction. There was sparse perivascular lymphomononuclear cell infiltrate in the dermis.

Skin biopsy was consistent with DM (Figure 2). Cervical biopsy showed keratinizing squamous cell carcinoma (SCC). CT scan of pelvis showed heterogeneous enhancing mass (69 × 89 mm) in the region of cervix and proximal part of vagina and distal part of body of uterus. Few soft tissue density lesions at both iliac fossa regions were seen. Chest X-ray and bone scintigraphy were normal. Thus, a diagnosis of keratinizing SCC uterine cervix stage III A with paraneoplastic DM was made for which she was started on PTF (Paclitaxel, Cisplatin, 5-Fluorouracil) chemotherapy followed by external beam radiation therapy. She was started on oral steroids (prednisolone 1 mg/kg), topical steroids, antihistamines, emollients, sunscreens, and physiotherapy for the treatment of dermatomyositis. After 6 weeks of treatment, she reported improvement in her muscle power without much improvement in dermatological manifestations. She also developed anagen effluvium secondary to chemotherapy and prominent follicular pluggings on scalp and forehead. After that, the patient was lost to follow-up.

## 3. Discussion

Dermatomyositis is a rare idiopathic inflammatory myopathy (IIM). It occurs at least twice as frequently in females as in males. In adults, onset is predominantly between the ages of 40 and 60 years. The mean age of onset is later in men than in women [3]. Paraneoplastic DM generally occurs in middle to elderly age group.

As malignancy in our case was diagnosed at such an advanced stage, onset of the malignancy must be few years back, and onset of DM was about 2 years back; these two events can be correlated temporally and DM occurring as paraneoplastic syndrome can, thus, be justified.

A model of crossover immunity for cancer-associated myositis has been suggested recently [4–6]. Common antigenic myositis-specific autoantigens expressed in both tumor cells and undifferentiated myoblasts lead to the generation of both specific T cells and B cells against those antigens and then to successful antitumor immunity. In a subset of patients, subsequent muscle damage from a variety of causes (such as viral infection and trauma) may lead to muscle damage and regeneration and may reactivate immune responses previously generated in the antitumor response. The crossover immunity between tumor cells and myofibroblasts may explain the parallel clinical course of both diseases [7].

A variety of investigations are available for the work-up of DM.

Serum creatine kinase (CK) level is usually the first step. About 80% to 90% of adult myositis patients show an increase in CK, but our patient had normal CK. Although muscle enzymes are frequently elevated in DM, they can be normal even in active disease with myositis [8]. Normal CK is relatively more common in DM than in PM [9]. Hence, measurement of other serum muscle enzymes, including aldolase, aspartate transaminase, alanine transaminase, and lactate dehydrogenase (LDH), significantly improves the chance of diagnosing active myositis, like in our patient with elevated LDH. This patient had normal liver function tests which help to rule out liver as source of these elevated enzymes.

Histopathologic findings of skin in DM are hyperkeratosis, vacuolization of the basal keratinocytes, melanin incontinence, perivascular lymphocytic infiltrate, and epidermal atrophy. However, histopathologic features are shared among DM and SLE. So, histopathology should be used to support the clinical diagnosis rather than being utilized as a diagnostic test.

Muscle biopsy remains the “gold standard” for the diagnosis of inflammatory myopathies such as DM. The features specific to DM include loss of capillaries, altered morphology of capillaries, capillary necrosis, complement deposition in the vessel walls and, rarely, muscle infarcts, and perifascicular atrophy. Bohan-Peter diagnostic criteria do not require muscle biopsy as a must to do investigation. Also, it is given the same weight as the other clinical criteria. So, in our patient diagnosis of DM can be justified on the basis of other findings as we were not able to do a muscle biopsy.

Electromyogram (EMG) changes are usually nonspecific but can serve as useful indicator of myopathic changes, to monitor disease activity and practical guide for biopsy sampling.

Identification of specific autoantibody strengthens the diagnosis. ANA has a low specificity for DM and has a sensitivity of 40–60%. Our patient had negative ANA. Autoantibodies in DM are now categorized into two groups: (1) myositis-specific autoantibodies which include Jo-1, PL-7, PL-12, OJ, Mi-2, and signal-recognition particle; (2) myositis-associated autoantibodies such as PM/Scl, Ro/SSA, and U1RNP.

The course is variable. Patients without muscle involvement have a better prognosis [10]. The overall mortality is approximately one-quarter [11]. Death usually occurs from respiratory infection, cardiac failure, and malnutrition due to difficulty in swallowing, malignancy, or from the side effects of therapy. The worst prognosis is seen in cancer-associated DM. The underlying malignancy, not the DM, accounts for the poor outcome. Removal of an underlying carcinoma in adults can lead to regression of the dermatomyositis [12]. Relapse of underlying neoplasm may be associated with exacerbation of DM.

## 4. Conclusion

This case report highlights the occurrence of DM as a paraneoplastic manifestation of cervical carcinoma with which it is rarely associated. Marked poikilodermatous changes on both upper and lower limbs, failure of full therapeutic response to conventional treatment of DM, and absence of elevation of usual muscle enzymes like creatine kinase were the unusual features in our case.

## Abbreviations

DM: Dermatomyositis
SLE: Systemic lupus erythematosus
SCC: Squamous cell carcinoma
CK: Creatine kinase
PM: Polymyositis
IIM: Idiopathic inflammatory myositis
ANA: Antinuclear antibodies.
